# Extracellular Vesicles Derived From *Entamoeba histolytica* Have an Immunomodulatory Effect on THP-1 Macrophages

**DOI:** 10.1155/2024/7325606

**Published:** 2024-10-29

**Authors:** Debabrata Chowdhury, Manu Sharma, James W. S. Jahng, Upinder Singh

**Affiliations:** ^1^Division of Infectious Diseases, Stanford University School of Medicine, Stanford, California 94305, USA; ^2^Stanford Cardiovascular Institute, Stanford University School of Medicine, Stanford, California 94305, USA; ^3^Department of Microbiology and Immunology, Stanford University School of Medicine, Stanford, California 94305, USA

**Keywords:** *E. histolytica*, extracellular vesicle, immune response, macrophage

## Abstract

Recent studies have shown that extracellular vesicles (EVs) secreted by various parasites are capable of modulating the host's innate immune responses, such as by altering macrophage (M*ϕ*) phenotypes and functions. Studies have shown that M*ϕ* promote early host responses to amoebic infection by releasing proinflammatory cytokines that are crucial to combating amoebiasis. Here, we are reporting for the first time the effect of EVs released by *Entamoeba histolytica* (*Eh*EVs) on human THP-1 differentiated M*ϕ* (THP-1 M*ϕ*). We show that the *Eh*EVs are internalized by THP-1 M*ϕ* which leads to differential regulation of various cytokines associated with both M1 and M2 M*ϕ*. We also saw that *Eh*EV treatment thwarted Type 2 immune-response-related transcriptome pSTAT6 in the THP-1 M*ϕ*. Furthermore, *Eh*EVs stimulated M*ϕ* to reduce their energy demand by suppressing oxidative phosphorylation (OXPHOS) and adenosine triphosphate (ATP) production. Hence, the human parasite *E. histolytica*–derived EVs are capable of eliciting an immune response from M*ϕ* that may contribute to overall infection status.

## 1. Introduction

The human protozoan parasite *Entamoeba histolytica* is the causative agent for intestinal amebiasis, leading to colitis in severe cases. It is estimated that *E. histolytica* infects around 10% of the total population worldwide. Although 90% of the infections are asymptomatic, around 100,000 deaths occur annually from approximately 50 million symptomatic patients, making amoebiasis the second leading parasitic cause of death worldwide [1]. During infection, amoeba use several mechanisms to avoid the host immune response for survival including interacting with surrounding microbiota for protective needs and using resources from the infected host for their persistence [2]. Therefore, identifying the host immune response to amoeba and the strategies developed by this parasite to escape immunity is critical to understanding disease pathogenesis.

Macrophages (M*ϕ*), neutrophils, and eosinophils are central in cell-mediated innate immunity to *E. histolytica* infection. M*ϕ* promote early host response to amoebic infection by releasing proinflammatory cytokines and recruiting other immune cells [3–5]. Lipopeptidophosphoglycan (LPPG) on the surface of amoeba and amoebic DNA is recognized by toll-like receptor (TLR) 2/4/6 and TLR 9, respectively, to induce a strong host immune response [6, 7]. The human primary peripheral blood mononuclear cells (PBMCs) were frequently employed to study immune modulation, but due to variations in cells obtained from different donors and technical inconsistencies associated with their handling, the human leukemia monocytic THP-1 cell line is widely accepted in vitro model [8, 9]. THP-1 monocytes differentiated into M*ϕ* using phorbol-12-myristate-13-acetate (PMA) markedly resemble PBMCs-derived M*ϕ* in metabolic, morphological, and functional properties [8–13].

Extracellular vesicles (EVs) are a heterogeneous group of particles secreted by both prokaryotic and eukaryotic cells and responsible for several physiological/pathological functions including intra- and intercellular communication. Various studies have reported that biomacromolecules packaged in EVs derived from parasites or parasite-infected host cells regulate host immune responses [14, 15].

Parasite-derived EVs can benefit the microbes by either dampening the innate immune response or by improving pathogen attachment to the host. For example, EVs from highly adherent strains of *Trichomonas vaginalis* augment the attachment of poorly adherent strains to human cervical epithelial cells [15]. In contrast, parasitic EVs can promote host immunity. For example, the *Toxoplasma gondii* exosome elevates proinflamatory IL-12, TNF*α*, and interferon gamma (IFN*γ*) but dampens immunosuppressive IL-10 in M*ϕ* [16].

Assessing the role of *Eh*EVs in the host is crucial to understanding the host immune response to *Entamoeba* infection. In this study, we show that *Eh*EVs are internalized by uncommitted (M0) THP-1 M*ϕ*, leading to differentially regulated cytokines related to the M1 and M2 M*ϕ* phenotypes. *Eh*EVs can also alter Type 2 responsiveness in THP-1 M*ϕ*. Moreover, *Eh*EVs are able to change the metabolic profile of M0 THP-1 M*ϕ*.

## 2. Materials and Methods

### 2.1. THP-1 Differentiation and Polarization

Human THP-1 leukemia monocytic cells were kindly provided by Prof. Paul L. Bollyky, Stanford University. THP-1 cells were differentiated by 24 h incubation with 150 nM PMA (Sigma, P8139-1MG) in RPMI medium (RPMI 1640, Corning; REF.: 10-041-CV) containing 10% heat-inactivated fetal bovine serum (FBS, HyClone; Cat. No.: SH30396.03) and supplemented with 50 pM *β*-mercaptoethanol (Sigma; Cat. No.: M7522-100 mL) and 1X Pen Strep (Gibco; REF.: 15-140-122). After 24 h, the PMA-containing medium was changed to a fresh RPMI medium. THP-1 M*ϕ* were stimulated with 5 ng/mL IFN*γ* (R&D Systems, Cat. No.: 285-IF-100/CF) for 24 h and 20 ng/mL IL-4 (PeproTech, Cat. No.: 200-04) for 1 h.

### 2.2. Fluorescence Microscopy

THP-1 M*ϕ* (250,000 cells/well) were seeded on an 8-well glass slide (Lab-TekII Chamber Slide, Cat. No.: 154534). Undifferentiated cells were attached to wells by drying with Dulbecco's phosphate-buffered saline (DPBS). Monocytes and M*ϕ* were fixed with 4% paraformaldehyde (Ted Pella, Inc., CA, Product No. 18505) diluted in DPBS and washed three times with ice-cold DPBS. To block the nonspecific binding of the antibodies, cells were incubated with 1% bovine serum albumin (BSA) and 22.52 mg/mL glycine in PBST (DPBS +0.1% Tween 20) for 1 h. The cells were washed three times with ice-cold DPBS and incubated with the primary antibodies: anti-CD (cluster of differentiation)14 (Novus Biologicals, Cat. No.: NBP2-37291), anti-CD36 (Novus Biologicals, Cat. No.: NB400-145SS), anti-CD68 (Novus Biologicals, Cat. No.: NB100-683), and anti-CD71 (ABclonal, Cat. No.: A5865) 1:200 diluted in 1% BSA in PBST overnight at 4°C. Cells were washed three times for 5 min with ice-cold DPBS. Cells were incubated with 2 *μ*g/mL Alexa Fluor-594-conjugated anti-rabbit IgG antibody (Invitrogen, Cat. No.: A11012) for 1 h in the dark at room temperature. Cells were washed three times for 5 min each with ice-cold DPBS and mounted with DAPI (Vector Laboratories Inc., Cat. No.: H-1200). Cells were observed with a fluorescent microscope (EVOS M5000 Imaging System—Invitrogen) under a 40X objective.

### 2.3. *E. histolytica* Culture and EV Purification

Parasites were grown to around 90% confluency in TYI medium (peptone: 34.29 g/L, glucose: 11.43 g/L, NaCl: 2.29 g/L, K_2_HPO_4_: 1.14 g/L, KH_2_PO_4_: 0.69 g/L, and cysteine: 1.29 g/L) supplemented with 15% heat-inactivated bovine serum, adult (Sigma, Cat. No.: B9433-500ML), 1X Diamond Vitamins (Gibco, Cat. No.: RR110017L1), and 1X Pen Strep in 25 cm^2^ culture flasks (Corning, REF.: 353014). Cells were washed twice with 5 mL of serum-free TYI complete medium. Then, the cells were incubated with 7.5 mL/flask serum-free medium for 24 h in an anaerobic chamber BD GasPak EZ Campy Container (BD, Cat. No.: 260680) with BD GasPak EZ Anaerobe Container System Sachets (BD, Cat. No.: 260678). The conditioned medium was collected and centrifuged at 600 rpm for 6 min at 4°C to remove cell debris. The supernatant was collected and centrifuged again at 2000 × g for 30 min at 4°C. For the isolation of EVs, the supernatant was ultracentrifuged at 100,000 × g for 18 h at 4°C using a high-speed ultracentrifuge (Beckman Optima L-70K, Cat. No.: 365678). The pellets were resuspended in 100 *μ*L of sterile DPBS.

To prepare the mock sample, all the above steps were carried out with a serum-free medium alone.

### 2.4. Nanoparticle Tracking Analysis (NTA)

The size of the EVs was measured by conventional and fluorescent-based NTA (fNTA) using Malvern's NanoSight LM10 NTA instrument (Particle Characterization Laboratories Inc., Novato, CA 94945).

For conventional NTA, 1 *μ*L of *Eh*EVs was diluted into 7.5 mL of filtered 1X PBS. For fNTA, 30 *μ*L of *Eh*EVs was labeled with ExoGlow-NTA Fluorescent Labeling Kit (SBI System Biosciences, Cat. No.: EXONTA200A-1), according to the manufacturer's instructions, with a final dye concentration of 28 *μ*M. Excess labeling dye was removed by a size exclusion column (PD SpinTrap G-25, Cytiva) before fNTA with an LM12 488 nm laser.

Each analysis was collected in triplicate for 90 s per repeat, amalgamated, and reported as a single combined result.

### 2.5. Internalization of EVs

*Eh*EVs were labeled with ExoGlow Protein EV Labeling Kit (SBI System Biosciences, Cat. No.: EXOGP100A-1) according to the manufacturer's instructions. Briefly, 20 *μ*L *Eh*EVs were diluted with 480 *μ*L DPBS, mixed with 1 *μ*L of 500X labeling dye, and incubated with shaking (350 rpm) for 20 mins at room temperature in the dark. ExoQuick-TC (167 *μ*L) was added to the mixture and incubated overnight at 4°C. The Eppendorf tube was centrifuged at 10,000 rpm for 10 min at 4°C. The supernatant was aspirated. The labeled *Eh*EV pellet was resuspended with 20 *μ*L DPBS and used for the uptake assay. A negative control was made with the same volume of mock samples labeled with the ExoGlow Protein EV Labeling Kit. THP-1 M*ϕ* (250,000 cells in 250 *μ*L of Opti-MEM medium) were exposed to 20 *μ*L of either labeled *Eh*EVs or mock for 24 h. Cells were fixed, blocked, permeabilized as before, and stained with primary antibody for anti-*β*-actin (ABclonal, Cat. No.: AC026) and 6 *μ*g/mL Alexa Fluor-488-conjugated anti-rabbit IgG antibody (Invitrogen, Cat. No.: A11034). Cells were observed with an Inverted Zeiss LSM880 laser scanning confocal microscope (Cell Sciences Imaging Facility: Beckman Center at Stanford University) under 63X objective using immersion oil.

### 2.6. Immunoassay

THP-1 M*ϕ* (250,000 cells in 250 *μ*L of Opti-MEM medium) were stimulated with either 10 *μ*L mock or *Eh*EVs in a 48-well tissue culture plate (Costar, Cat. No.: 3548) for 24 h. Cell-free supernatant was collected by centrifugation at 600 rpm for 6 min at 4°C. The centrifugation step was repeated once again, and the supernatant was collected and stored on ice without freezing until the immune assay on the same day. Cytokines associated with M*ϕ* activity were analyzed using a Luminex Human 80 plex assay custom combination from EMD Millipore panels in the Human Immune Monitoring Center at Stanford University.

### 2.7. Viability Assay

One hundred twenty-five thousand THP-1 cells were seeded with 125 *μ*L of Opti-MEM medium per well of a 96-well plate and stimulated with either 5 *μ*L of mock or *Eh*EVs for 24 and 48 h. Paraformaldehyde (2%) treatment for 30 min was used as cell death control. Then the cells were incubated with 40 *μ*M of resazurin (Sigma-Aldrich, Cat. No.: R7017) for 1 h at 37°C. Plates were read at 595 nm after 535 nm excitation by GloMax Discover Microplate Reader (Promega, Cat. No.: GM3000) according to the manufacturer's instructions.

### 2.8. Western Blots

For THP-1 differentiation, anti-CD14 (Novus Biologicals, Cat. No.: NBP2-37291), anti-CD36 (Novus Biologicals, Cat. No.: NB400-145SS), anti-CD68 (Novus Biologicals, Cat. No.: NB100-683), anti-CD71 (ABclonal, Cat. No.: A5865), and anti-*β*-actin (ABclonal, Cat. No.: AC026) were used to analyze protein levels in the total cell lysate. Lectin light chain (Lgl) and pan-actin were measured in the whole cell lysate of *E. histolytica* and the parasite-derived *Eh*EVs using 1:10,000 anti-Lgl (kindly gifted from Prof. William Petri Jr, School of Medicine, University of Virginia) and anti-pan-actin (Cell Signaling Technology, Cat. No.: 4968S), respectively. Anti-pSTAT1 (Cell Signaling Technology, Cat. No.: 7649), anti-pSTAT6 (Cell Signaling Technology, Cat. No.: 9361), and anti-*β*-actin were used to assay protein level in whole cell lysate of THP-1 M*ϕ* (250,000 cells in 250 *μ*L of Opti-MEM medium in a 48-well plate). For pSTAT1, THP-1 M*ϕ* were cocultured with either 10 *μ*L mock or *Eh*EVs with hIFN*γ* (5 ng/mL) for 24 h. For pSTAT6, M*ϕ* were stimulated for 23 h with either 10 *μ*L mock or *Eh*EVs prior to the treatment with IL-4 (20 ng/mL) for 1 h (incubation time was determined based on the highest pSTAT6 signal). Total cell lysate was prepared using Cell Culture Lysis Buffer 5X Reagent (Promega, REF.: E153A) containing 1X Halt Protease Inhibitor Cocktail, EDTA-free (ThermoScientific, Cat. No.: 78425), 1 mM Leupeptin (ThermoScientific, Cat. No.: 78435) and 1 mM E-64 (ThermoScientific, Cat. No.: 78434). Protein concentration was measured using Protein Assay Dye Reagent (Bio-Rad, Cat. No.: 5000006). Equal amounts of proteins were run on 10% SDS-PAGE. Proteins were transferred onto Immun-Blot PVDF Membranes for Protein Blotting (Bio-Rad, Cat. No.: 1620177). Western blots were blocked with 5% BSA (Sigma, Cat. No.: A4503-11G) 1X TBST (Tris: 20 mM, NaCl: 150 mM, Tween 20 detergent: 0.1% *v*/*v*, and pH: 7.4) and probed with primary antibodies and their corresponding host-specific HRP-conjugated secondary antibodies and developed using ECL Prime Western Blotting Detection Reagent (Cytiva, Cat. No.: RPN2236) on X-ray Film (ThermoScientific, REF.: 34091).

### 2.9. Flow Cytometry

THP-1 M*ϕ* (250,000 cells in 250 *μ*L of Opti-MEM medium in a 48-well plate) were treated with either 10 *μ*L mock or *Eh*EVs for 48 h. M*ϕ* were stained with MitoTracker Deep Red (Invitrogen; Cat No. M22462 for mitochondrial mass) as per the manufacturer's instructions, and cells were fixed with 4% paraformaldehyde. Fixed cells were analyzed by the Cytek Aurora Northern Lights flow cytometer. Data were analyzed using FlowJO software; data were presented as percentages of mean fluorescent intensity.

#### 2.9.1. Metabolic Assays

THP-1 M*ϕ* were seeded in an XF96 seahorse cell culture microplate at 50,000 cells/well with 100 *μ*L of Opti-MEM medium. Six technical replicates were used for each experimental group. M*ϕ* were treated with either 2 *μ*L mock or *Eh*EVs/well for 48 h. On the day of measurement, the media was replaced with unbuffered DMEM supplemented with glucose (10 mM final), glutamine (2 mM final), and pyruvate (1 mM final) and adjusted to a pH of 7.4. Cells were equilibrated at 37°C in a CO_2_-free incubator for 15 min. Inhibitors (oligomycin and rotenone/antimycin) were added at a final concentration of 2 and 1 *μ*M, respectively, after injection to the appropriate port of the sensor cartridge. The cell culture plate and sensor cartridge were run on the Seahorse XF-96, and data were analyzed by Excel.

## 3. Results

### 3.1. *E. histolytica* Parasite-Derived EVs Are Internalized by THP-1 M*ϕ*

Our lab had previously characterized *Eh*EVs that were isolated using a PEG-based commercial kit [17]. Since this method might lead to contaminations with kit components that could potentially interfere with the immune response, we used an ultracentrifugation-based protocol and recharacterized the isolated *Eh*EVs for the present study [18].

**Figure 1:**
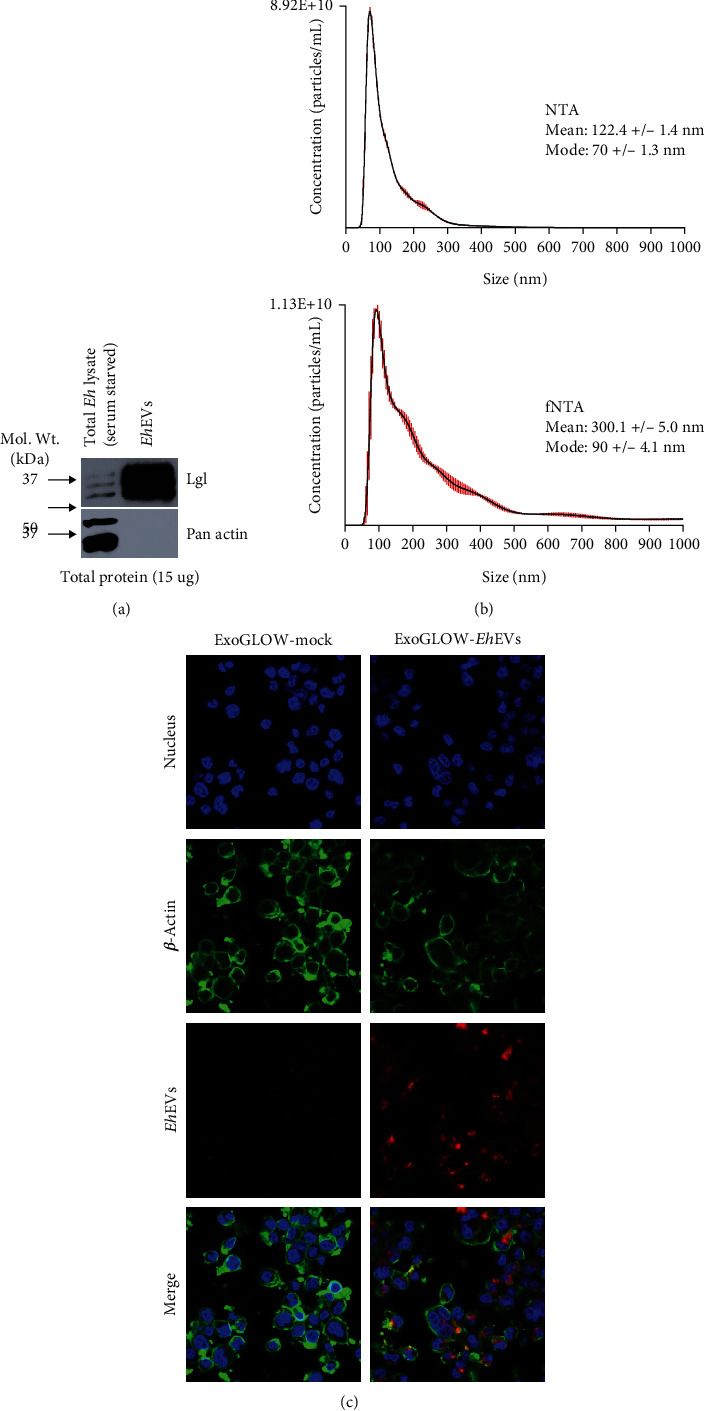
Characterization of *E. histolytica*–derived EVs and their internalization into the THP-1 M*ϕ*. (a) Western blots of the membrane protein light chain of galactose/*N*-acetylgalactosamine lectin (Lgl) and cytoskeleton protein actin in 15 *μ*g protein of *Eh*EVs and total cell lysate of *E. histolytica* after releasing *Eh*EVs. (b) Size distribution of *Eh*EVs measured with conventional nanoparticle tracking analysis (NTA) and fluorescent-based NTA (fNTA). Data were averaged from three individual runs of the same sample and presented as finite track-length adjusted (FTLA) concentration. (c) Confocal microscopy images represent the *Eh*EV uptake by THP-1 M*ϕ*. Cells (250,000 cells in each well of an eight-well chamber) were incubated for 24 h with 20 *μ*L of mock or *Eh*EVs labeled with ExoGLOW (red). *β*-Actin (green) and DAPI (blue) were used to distinguish the subcellular localization of *Eh*EVs in the cells.

The membrane protein—Gal/GalNAc Lgl—has been shown to be highly enriched in *Eh*EVs, while the cytoskeleton protein—actin—is depleted in *Eh*EVs in comparison with the total cell lysate. As reported previously, we found that Lgl was selectively enriched and pan-actin was highly depleted in *Eh*EVs prepared by ultracentrifugation (in comparison with total cell lysate) (Figure 1(a)) [17]. Conventional NTA and fNTA (using an EV-specific dye) showed that the mean size of *Eh*EVs is 122 and 300 nm, respectively (Figure 1(b)). For a better understanding of the differences in the purified *Eh*EV size, the overlay image of conventional and fluorescence-based NTA is illustrated in Supporting Information 1.

We next investigated if THP-1 M*ϕ* were efficiently internalizing the *Eh*EVs. THP-1 monocytic cells were first differentiated into THP-1 M*ϕ* using PMA. This was confirmed using microscopy since the THP-1 M*ϕ* population is adherent and therefore morphologically distinct from the undifferentiated THP-1 monocytes (nonadherent, suspension cell line) (Supporting Information 1). The monocyte to M*ϕ* differentiation markers—CD (cluster of differentiation)14, CD36, CD68, and CD71—were also analyzed by immunoblotting and immunofluorescence-based imaging (Supporting Information 1). CD14 expression was decreased after the differentiation of THP-1 cells (Supporting Information 1). The monocyte differentiation into the M*ϕ* was also confirmed by analyzing the expression of CD36, CD68, and CD71, which were increased similar to earlier reports (Supporting Information 1) [19–22]. Next, mock and *Eh*EVs were stained with the red fluorescent dye, ExoGLOW, and the uptake was assayed using confocal microscopy. After 24 h treatment of THP-1 M*ϕ* with ExoGLOW-mock or ExoGLOW-*Eh*EVs, only ExoGLOW-*Eh*EVs-treated M*ϕ* showed intense red fluorescence in their cytoplasm but not nuclear compartment (Figure 1(c)).

### 3.2. EVs Released by *E. histolytica* Do Not Affect THP-1 M*ϕ* Viability

**Figure 2:**
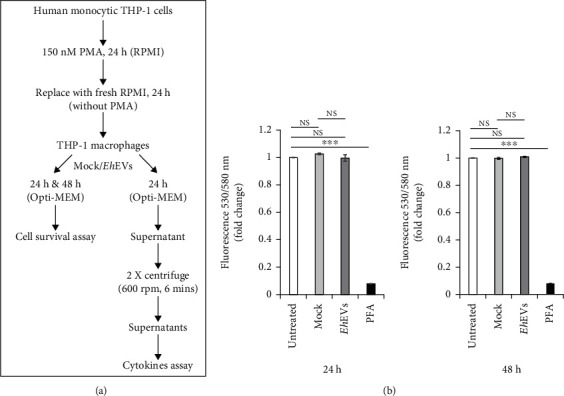
*E. histolytica*–derived EV effect on the viability of THP-1 M*ϕ*. (a) Schematic representation of the method used for assaying THP-1 M*ϕ* viability and cytokine profiling. (b) Viability was measured at 24 and 48 h of 5 *μ*L of either mock or *Eh*EVs stimulated 125,000 THP-1 M*ϕ* in 125 *μ*L Opti-MEM medium. Paraformaldehyde (2% PFA, 30 min) was used as a positive control for cell death. Bar graphs represent fold changes, one-way ANOVA (Holm–Sidak method), four independent experiments, data are mean ± SEM. NS, not significant.

We next checked if the internalization of *Eh*EVs in the THP-1 M*ϕ* affected their viability. THP-1 M*ϕ* were exposed to mock or *Eh*EVs for 24 and 48 h and compared with resting M*ϕ* or M*ϕ* undergoing cell death. To induce cell death as a positive control for this study, cells were treated with 2% paraformaldehyde for 30 min. It was observed that exposure to *Eh*EVs did not alter cell viability (Figures 2(a) and 2(b)).

### 3.3. EVs Released by *E. histolytica* Elicit a Complex Immune-Response in THP-1 M*ϕ*

**Figure 3:**
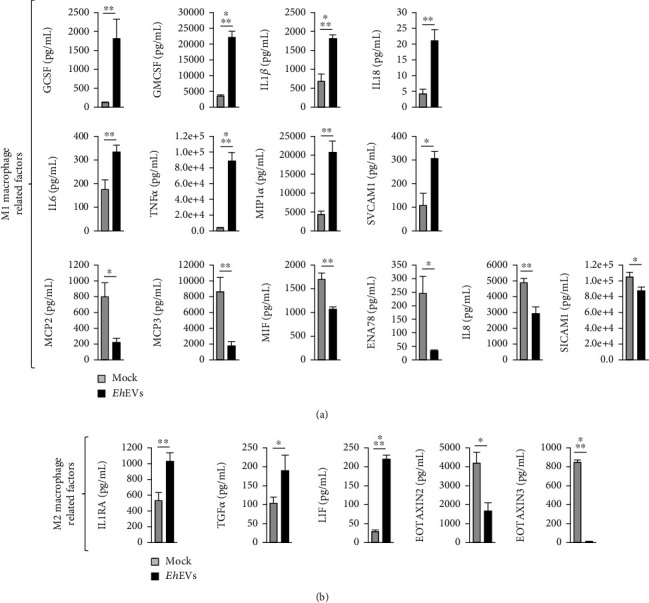
*E. histolytica*–derived EVs differentially regulate M1 and M2 THP-1 M*ϕ*-related cytokines. THP-1 M*ϕ* (250,000 cells in 250 *μ*L of Opti-MEM medium) were stimulated for 24 h with either 10 *μ*L mock or *Eh*EVs. (a) M1 M*ϕ*-related cytokines: GCSF, GMCSF, IL-1*β*, IL-18, IL-6, TNF*α*, MIP1*α* or CCL3, SVCAM1, MCP2 or CCL8, MCP3 or CCL7, MIF, ENA78 or CXCL5, IL-8 or CXCL8, and SICAM1. (b) M2 M*ϕ*-related cytokines: IL-1RA, TGF*α*, LIF, EOTAXIN2 or MPIF2 or CCL24, and EOTAXIN3 or CCL26 concentration (picograms per milliliter) were analyzed using Luminex Human 80 plex assay custom combination from EMD Millipore panels, three to five independent experiments, two-tailed *t*-test, data are mean ± SEM: ⁣^∗^*p* < 0.05, ⁣^∗∗^*p* < 0.01, and ⁣^∗∗∗^*p* < 0.001.

Since *Eh*EVs were found to be internalized in the M*ϕ* at 24 h, we decided to check for cytokine secretion at this time point. Differential regulation of the cytokines associated with both M1 and M2 M*ϕ* phenotypes was observed in THP-1 M*ϕ* stimulated with *Eh*EVs for 24 h. The steps we followed to measure these protein modulators are schematically represented in Figure 2(a). We consistently observed the upregulation of certain M1 M*ϕ*–related factors and the downregulation of others (results show the mean of at least three and up to five independent studies) (Supporting Information 2). The M1 M*ϕ* related factors that were found to be significantly upregulated in the supernatant of THP-1 M*ϕ* upon *Eh*EV exposure, compared to mock control, were granulocyte colony-stimulating factor (GCSF), granulocyte-macrophage colony-stimulating factor (GMCSF), IL-1*β*, IL-18, IL-6, TNF*α*, macrophage inflammatory protein (MIP)1*α* or chemokine (C-C motif) ligand (CCL)3, and circulating vascular cell adhesion molecule (SVCAM)1 (Figure 3(a). The M1 M*ϕ*–related factors that were downregulated were monocyte chemotactic protein (MCP)2 or CCL8, MCP3 or CCL7, macrophage migration inhibitory factor (MIF), epithelial neutrophil-activating (ENA) protein 78 or chemokine (C-X-C motif) ligand (CXCL)5, IL-8 or CXCL8, and soluble intercellular adhesion molecule (SICAM)1 (Figure 3(a)).

Interestingly, certain M2 M*ϕ*–related factors were also found to be differentially regulated—IL-1 receptor antagonist (IL-1RA), transforming growth factor (TGF)*α*, and leukemia inhibitory factor (LIF) were found to be upregulated, and eosinophil chemotactic protein (EOTAXIN)2 or myeloid progenitor inhibitory factor (MPIF)2 or CCL24 and EOTAXIN3 or CCL26 were significantly downregulated (Figure 3(b)).

### 3.4. EVs Released by *E. histolytica* Modulate Type 2 Immune Response in THP-1 M*ϕ*

The proinflammatory Type 1 cytokine, IFN*γ*, polarizes M*ϕ* into M1 M*ϕ* phenotype by activation of pSTAT1, whereas Type 2 anti-inflammatory cytokine IL-4 elicits an M2 M*ϕ* phenotype via pSTAT6 activation [23]. As *Eh*EVs have a mixed regulatory effect on immune modulators associated with both M1 and M2 M*ϕ* phenotypes, we checked if *Eh*EVs control IL-4 and/or IFN*γ* responses in THP-1 M*ϕ*.

To investigate Type 1 immune response, THP-1 M*ϕ* were exposed to IFN*γ* alone or in combination with mock or *Eh*EVs, and pSTAT1 was analyzed by western blotting in the total cell lysate. However, *Eh*EVs treatment had no effect on pSTAT1 levels upon IFN*γ* treatment in the THP-1 M*ϕ* at 6 h (data not shown).

**Figure 4:**
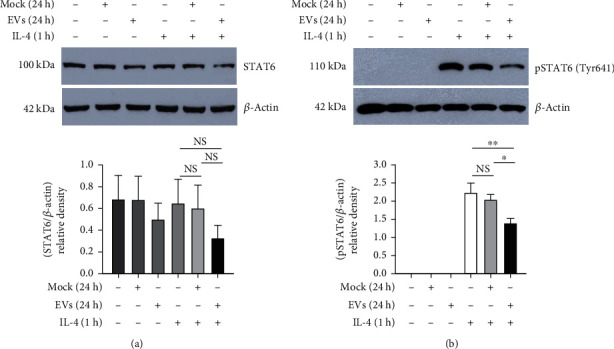
*E. histolytica*–derived EVs regulate the IL-4 response in THP-1 M*ϕ*. Western blots of pSTAT6 (downstream of IL-4) and *β*-actin in total lysates of 250,000 M*ϕ* stimulated with either 10 *μ*L mock or *Eh*EVs for 24 h. (a) STAT6 was analyzed in cell lysates from mock or *Eh*EVs were treated alone for 23 h prior to the addition of 20 ng/mL human IL-4 for another 1 h; bar graphs represent densitometric analysis normalized to *β*-actin, one-way ANOVA (Fisher's LSD method), three independent experiments, data are mean ± SEM. (b) For pSTAT6, the same samples were run on a separate western blot and probed with anti-pSTAT6 antibody; bar graphs represent densitometric analysis normalized to *β*-actin, one-way ANOVA (Holm–Sidak method), three independent experiments, data are mean ± SEM: ⁣^∗^*p* < 0.05 and ⁣^∗∗^*p* < 0.01. NS, not significant.

To explore the effect on Type 2 immune response, THP-1 M*ϕ* were prestimulated with mock control or *Eh*EVs for 23 h followed by exposure with anti-inflammatory IL-4 for 1 h (pSTAT6 signal was seen to be highest at 1 h post IL-4 treatment—data not provided). The total cell lysate was used to determine the pSTAT6 level by western blotting. STAT6 levels were also checked for the same samples in a separate western blot and found to be unaltered (Figure 4(a)). *Eh*EV treatment significantly reduced pSTAT6 in the IL-4-treated M*ϕ* compared to the mock control (Figure 4(b)).

### 3.5. EVs Released by *E. histolytica* Alter Metabolic Profile of THP-1 M*ϕ*

**Figure 5:**
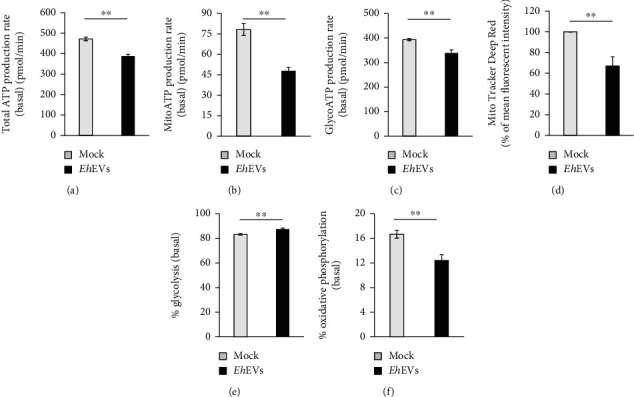
*E. histolytica*–derived EVs direct metabolism of THP-1 M*ϕ*. For metabolic analysis, THP-1 M*ϕ* (50,000 cells in 100 *μ*L of Opti-MEM medium) were seeded in each well of a Seahorse Biosciences XF48 cell culture microplate and stimulated for 48 h with either 2 *μ*L of mock or *Eh*EVs. At the basal level, (a) total ATP production rate, (b) MitoATP production rate, and (c) GlycoATP production rate were assayed using a Seahorse XFe96 Analyzer. Six technical replicates, three independent experiments, two-tailed *t*-test, data are mean ± SEM: ⁣^∗∗^*p* < 0.01. (d) THP-1 M*ϕ* (250,000 cells in 250 *μ*L of Opti-MEM medium) were stimulated with either 10 *μ*L mock or *Eh*EVs for 48 h and stained with MitoTracker Deep Red and analyzed by Cytek Aurora, Northern Lights flow cytometer. Data were analyzed using FlowJO software and presented as percent of mean fluorescent intensity. Six independent experiments, two-tailed *t*-test, data are mean ± SEM: ⁣^∗∗^*p* < 0.01. (e) For percent (%) of glycolysis and (f) percent of oxidative phosphorylation (% OXPHOS) analysis, THP-1 M*ϕ* were stimulated with mock or *Eh*EVs in Seahorse Biosciences XF48 cell culture microplate as above. At the basal level, % glycolysis and % OXPHOS were assayed using Seahorse XFe96 Analyzer. Six technical replicates, three independent experiments, two-tailed *t*-test, data are mean ± SEM: ⁣^∗∗^*p* < 0.01.

Since the *Eh*EVs were effectively altering the immune response of THP-1 M*ϕ*, we decided to further check the effect of *Eh*EVs on M*ϕ* phenotype. The metabolic state of the M*ϕ* has been closely linked to the immune response and shown to significantly influence its phenotype and function [24, 25]. We measured the energetic profile of the M*ϕ* after stimulating with *Eh*EVs using a Seahorse XF Real-Time ATP Rate Assay Kit. After stimulating with *Eh*EVs for 48 h, THP-1 M*ϕ* suppressed the total ATP production rate (Figure 5(a)). Total ATP production from oxidative phosphorylation (OXPHOS) and glycolysis significantly decreased (Figures 5(b) and 5(c)). Mitochondrial staining (MitoTracker Deep Red) confirmed that mitochondrial contents significantly decreased in THP-1 M*ϕ* stimulated with *Eh*EVs for 48 h (Figure 5(d)). Although the overall energy production rate from both OXPHOS and glycolysis decreased after stimulation with *Eh*EVs, the relative energy production rate specifically from glycolysis increased, suggesting that *Eh*EVs potentially skew energy utilization of THP-1 M*ϕ* towards glycolysis (Figures 5(e) and 5(f)).

## 4. Discussion

Our study evaluates the immune response to *Eh*EVs in human THP-1 M*ϕ*. We first optimized EV isolation using an ultracentrifugation-based protocol. In our previous work on characterizing *Eh*EVs, we used a PEG-based protocol [17]. However, to avoid any interference in the immune response from the PEG-based kit components, we employed an ultracentrifugation-based protocol to isolate EVs. These EVs carried similar protein markers as the previously characterized EVs (overexpression of Lgl and depletion of pan-actin). We characterized the EVs using both conventional NTA and fNTA. fNTA only detects particles labeled with an EV-specific dye, which may account for the lower concentration observed compared to conventional NTA. A marginal discrepancy between the size measurements obtained from the two methods was noted, as seen in the overlay in Supporting Information 1. The size distribution for the fNTA was slightly higher (right-shifted) compared to the NTA distribution. The mode for NTA was 70 nm compared to 90 nm for fNTA. We did not observe any unique population in NTA that was missing in fNTA, suggesting that the presence of unlabeled cell debris (not labeled with EV-specific dye) is unlikely to be the cause of the discrepancy. The slightly larger size could be because of technical issues with the fNTA as reported in previous studies [26]. It is possible that smaller particle sizes were lost during the staining protocol, or these were not effectively stained and therefore not detected evenly by the fNTA. The secretion of both pro- and anti-inflammatory cytokines by M*ϕ* upon stimulation with EVs from other organisms has been reported before [27, 28]. Both types of cytokines were found to be differentially regulated depending on the concentration and time of *Leishmania shawi* and *Leishmania guyanensis* EVs exposure to the M*ϕ* [29]. Similarly, we found that *Eh*EVs elicit a mixture of pro- and anti-inflammatory responses. A mixed M*ϕ* response indicates the *Eh*EVs carry a complex cargo of immunogenic effectors capable of modulating the immune cells in different ways. It is also possible that heterogenous EVs were collected from the heterogenous population of parasites, resulting in a mixed response. Additionally, the EV preparation might have included cellular components such as protein aggregates or small membrane fragments from dead cells, despite our efforts to minimize this using a sequential centrifugation protocol. Nevertheless, the presence of such contaminants cannot be completely ruled out and may contribute to the observed heterogeneity. Or else, it is possible that individual *Eh*EVs carried both pro- and anti-inflammatory molecules. Studies reported that the interaction of LPPG (exposed on the cell surface of *E. histolytica*) with TLR2 on peritoneal monocytes and M*ϕ* releases TNF*α*, IL-6, IL-8, IL-12, and IL-10 via activation of NF-*κβ* [7]. Moreover, Gal/GalNAc lectin, another major *E. histolytica* cell surface component, is also able to activate NF-*κβ*, leading to the transcription of TLR2 [30]. Our previous study found that *Eh*EVs contain both LPPG and Gal/GalNAc lectin, and these factors may be responsible for the induction of both pro- and anti-inflammatory cytokines [17]. MCPs and IL-8 are known to be immune cell chemoattractant factors that recruit immune cells to the site of infections [2, 31, 32]. We show here that MCP2, MCP3, and IL-8 levels are reduced by *Eh*EVs, suggesting that they can facilitate infection. Eosinophil-attracting chemokines such as eotaxin-1/CCL11, eotaxin-2/CCL24, and eotaxin-3/CCL26 promote host immunity against parasitic and bacterial infection [33, 34]. We found that the *Eh*EVs dampen eotaxin-2/CCL24 and eotaxin-3/CCL26 expression, which also indicates that the EVs can favor parasite survival.

Activation of M*ϕ* during infection is complicated and context-dependent. M1 M*ϕ* promote inflammation and facilitate pathogen killing, while M2 M*ϕ* are mainly involved in tissue-repairing processes [35–37]. Interestingly, it was seen that the Type 2 cytokine IL-4 is rapidly upregulated and sustained during intracecal amebic infection, whereas the Type 1 cytokine IFN*γ* is suppressed [38]. IL-4 and IL-13 play a critical role in allergic inflammation and parasite infection and act through the STAT6 signaling pathway [39]. In this study, we found that THP-1 M*ϕ* stimulated with *Eh*EVs thwart the IL-4 response by suppressing pSTAT6. More work needs to be done to understand the effect of *Eh*EVs on macrophage polarization.

The metabolic profile of M1 M*ϕ* is distinct from M2 M*ϕ*. For example, M1 M*ϕ* utilize mainly glycolysis, whereas M2 M*ϕ* mostly prefer OXPHOS for their energy demand [23]. It is now well-established that immunometabolism directs immune cell function, and this has been studied in detail [40–42]. For example, metabolic reprogramming of immune cells is exploited by parasites to manipulate the immune cell activation that controls parasitism. *Trypanosoma cruzi*–infected M*ϕ* have reduced levels of glycolysis and oxidative species compared to M1 M*ϕ* [43]. Moreover, EVs are now recognized as efficient messengers for intercellular communication to regulate target genes for reprogramming the recipient cell metabolism and their function [44, 45]. We have observed that *Eh*EV treatment altered the energetics in THP-1 M*ϕ*. We believe that the decreased energy production by *Eh*EVs in THP-1 M*ϕ* happens primarily via suppression of OXPHOS and mitochondrial content.

## 5. Conclusion

Altogether, our investigations demonstrate that *Eh*EVs act as a delivery vehicle of immunologically active biomacromolecules which can contribute to host–pathogen interaction. Our previous findings reported that *Eh*EVs have a complex molecular composition, and it was not surprising to find that these EVs elicit a mixture of immune responses in THP-1 M*ϕ*. *Eh*EVs could have a role in influencing M*ϕ* phenotype and function and, consequently, in the inflammatory processes associated with *E. histolytica* infection. Our discovery raises important questions to investigate whether *Eh*EVs are protective of the host or promote the pathogenesis of invasive amebiasis.

## Data Availability

The datasets are provided in this research article and discussed herewith to support the results and conclusion.

## Supporting Information

Supporting Information 1 Figure S1: size distribution of EhEVs measured with nanoparticle tracking analysis (NTA). Overlay image of size distribution of the purified EVs derived from *E. histolytica* with conventional and fluorescence-based NTA. Data were average from three individual runs of the same sample and presented as finite track-length adjusted (FTLA) concentration. Figure S2: differentiation of the human THP-1 monocytic cells into THP- 1 M*ϕ*. THP-1 cells were differentiated into M*ϕ* by 24 h incubation with complete RPMI medium containing 150 nM of phorbol 12-myristate 13-acetate (PMA), followed by 24 h in complete RPMI medium without PMA. (A) PMA-treated and untreated THP-1 cells were imaged with a phase contrast microscope. (B) Western blots were performed to measure expression of CD14, CD36, CD68, and CD71 protein levels in PMA-treated and untreated total THP-1 cell lysate. Bar graphs represent densitometric analysis normalized to *β*-actin; two-tailed *t*-test; three independent experiments; data are mean ± SEM: ⁣^∗∗^*p* < 0.01. (C–F) Undifferentiated and differentiated cells were fixed with 4% paraformaldehyde and immunolabeled for CD14, CD36, CD68, and CD71 using specific antibodies with Alexa Fluor-594-conjugated anti-rabbit IgG antibody (red). Nuclei were stained with 4⁣′,6-diamidino-2-phenylindole (DAPI, a blue-fluorescent DNA stain). Images were captured using a fluorescent microscope under a 40X magnification objective.

Supporting Information 2 Table S1. Raw data used for analyzing the cytokine profile.
