# Supplementary material for: Extracellular Vesicles Derived From Entamoeba histolytica Have an Immunomodulatory Effect on THP-1 Macrophages
Source: J Parasitol Res. 2024 Oct 29;2024:7325606. doi: 10.1155/2024/7325606 (PMC11537751; doi:10.1155/2024/7325606)
Supplement: Supporting Information 1 — Figure S1: size distribution of EhEVs measured with nanoparticle tracking analysis (NTA). Overlay image of size distribution of the purified EVs derived from E. histolytica with conventional and fluorescence-based NTA. Data were average from three individual runs of the same sample and presented as finite track-length adjusted (FTLA) concentration. Figure S2: differentiation of the human THP-1 monocytic cells into THP- 1 Mϕ. THP-1 cells were differentiated into Mϕ by 24 h incubation with complete RPMI medium containing 150 nM of phorbol 12-myristate 13-acetate (PMA), followed by 24 h in complete RPMI medium without PMA. (A) PMA-treated and untreated THP-1 cells were imaged with a phase contrast microscope. (B) Western blots were performed to measure expression of CD14, CD36, CD68, and CD71 protein levels in PMA-treated and untreated total THP-1 cell lysate. Bar graphs represent densitometric analysis normalized to β-actin; two-tailed t-test; three independent experiments; data are mean ± SEM: ⁣∗∗p < 0.01. (C–F) Undifferentiated and differentiated cells were fixed with 4% paraformaldehyde and immunolabeled for CD14, CD36, CD68, and CD71 using specific antibodies with Alexa Fluor-594-conjugated anti-rabbit IgG antibody (red). Nuclei were stained with 4⁣′,6-diamidino-2-phenylindole (DAPI, a blue-fluorescent DNA stain). Images were captured using a fluorescent microscope under a 40X magnification objective. [file 7325606.f1.zip › Supplement figure S1.pptx]

## Slide 1
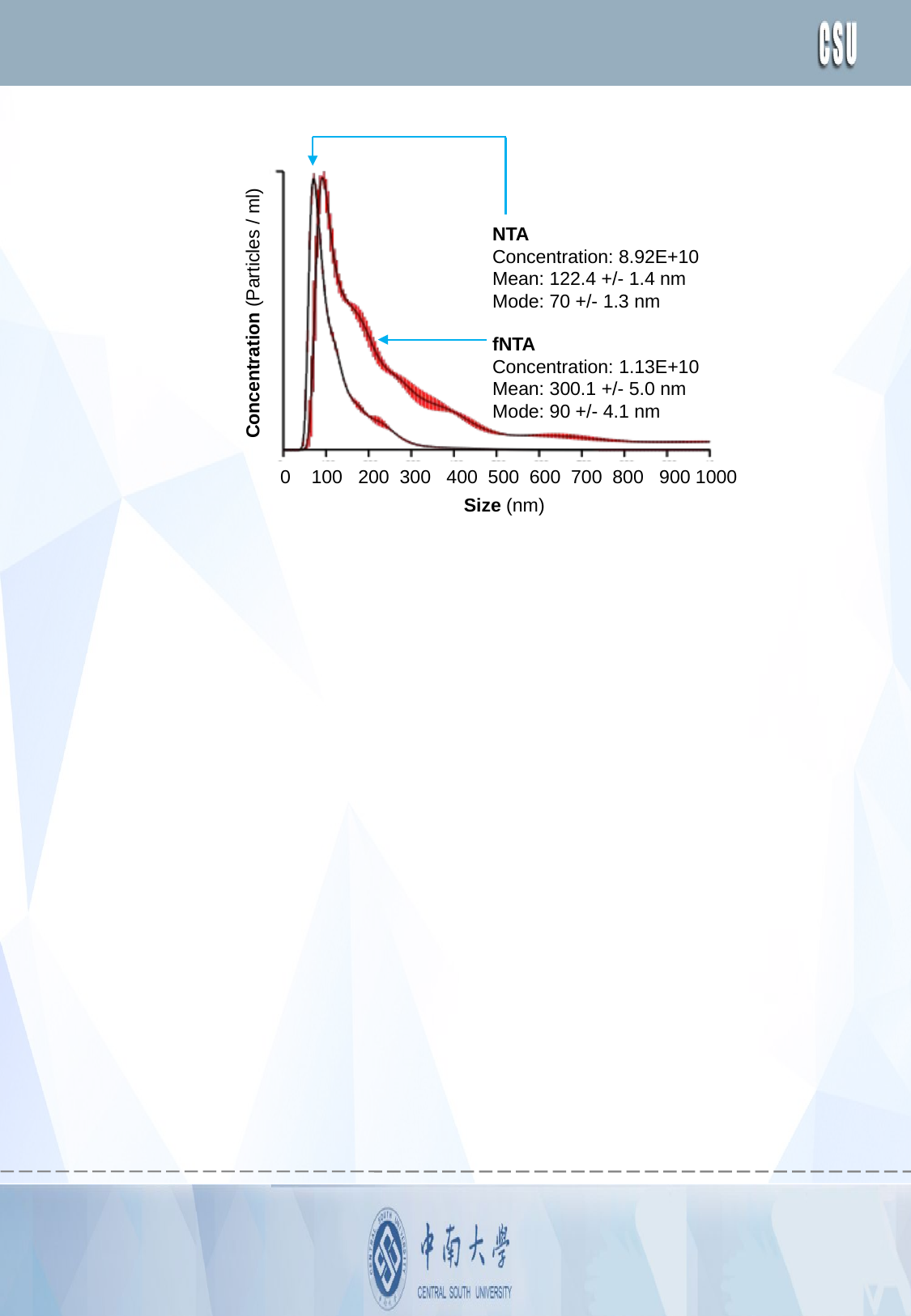

NTA
Concentration: 8.92E+10
Mean: 122.4 +/- 1.4 nm
Mode: 70 +/- 1.3 nm
Concentration (Particles / ml)
fNTA
Concentration: 1.13E+10
Mean: 300.1 +/- 5.0 nm
Mode: 90 +/- 4.1 nm
0 100 200 300 400 500 600 700 800 900 1000
Size (nm)
