# Supplementary material for: Extracellular Vesicles Derived From Entamoeba histolytica Have an Immunomodulatory Effect on THP-1 Macrophages
Source: J Parasitol Res. 2024 Oct 29;2024:7325606. doi: 10.1155/2024/7325606 (PMC11537751; doi:10.1155/2024/7325606)
Supplement: Supporting Information 2 — Table S1. Raw data used for analyzing the cytokine profile. [file 7325606.f2.pdf]

		SCD40L	EGF	EOTAXIN/CCL	FGF2/FGFB	FLT3L
Group Name	Wells	SCD40L pg/mL	EGF pg/mL	EOTAXIN/CCL11 pg/mL	FGF2/FGFB pg/mL	FLT3L pg/mL
SB	H1,	<12.80	<3.20	1.89	29.45	0.9
Mock (Ultra)	A8,	101.23	17.46	8.37	44.2	1.08
Mock (Ultra)	B8,	159.22	18.23	9.55	35.71	1.29
Eh EVs (Ultra)	C8,	149.54	16.39	22.56	37.19	0.9
EhEVs (Ultra)	D8,	146.27	17.98	23.71	35.71	1.34
CONS-4 DX3 (	G7,F7,	505.92	16.77	73.3	101.28	6.34
PBS	H7,	<12.80	10.82	<1.89	29.45	<0.90
S7	A1,	217027.73	38748.84	28091.52	346253.67	10251.28
S6	B1,	39605.11	10454.55	11581.08	69121.2	3407.46
S5	C1,	8625.16	2088.95	2260.64	18700.15	584.57
S4	D1,	1447.07	390.66	373.51	3005.08	119.55
S3	E1,	352.8	80.97	83.78	668.51	24.94
S2	F1,	86.11	15.83	15.71	125.33	4.64
S1	G1,	<12.80	3.38	3.25	26.05	0.98

FRACTALKINE	GCSF	GMCSF	GROA	IFNA2	IFNG	IL1A
FRACTALKINE/CX3CL1 pg/mL	GCSF pg/mL	GMCSF pg/mL	GROA pg/mL	IFNA2 pg/mL	IFNG pg/mL	IL1A pg/mL
31.96	<4.80	<2.06	<1.22	<8.00	<0.47	4.25
51.65	113.78	3086.18	12.47	<8.00	97.04	500.3
51.65	86.05	2753.4	12.79	<8.00	97.88	571.49
57.33	2494.79	25087.97	13.11	46.99	92.8	1329.75
101.52	3485.81	27279.25	27.45	96.12	91.24	1409.26
195.21	145.7	185.44	<1.22	<8.00	21.73	10.35
28.04	24.52	210.14	<1.22	<8.00	10.03	5.02
347975.38	81871.88	41669.62	13448.46	828833.09	20119.06	58385.49
117389.37	14454.82	7550.77	4675.58	23110.45	3967.11	17370.43
19514.95	3179.32	1688.75	780.46	5230.26	852.37	2796.43
4034.84	562.74	302.54	162.35	952.45	147.41	525.91
796.97	127.31	63.22	31.55	211.66	32.84	143.53
160.25	23.19	15.14	6.55	36.75	8.07	26.9
31.96	4.96	2.06	1.22	10.23	0.47	3.47

IL1B	IL1RA	IL2	IL3	IL4	IL5	IL6
IL1B	IL1RA	IL2	IL3	IL4	IL5	IL6
pg/mL	pg/mL	pg/mL	pg/mL	pg/mL	pg/mL	pg/mL
<1.25	<1.60	<0.41	0.21	<0.21	<0.05	0.22
224.42	305.39	2.93	3.95	33.28	0.05	98.18
177.53	254.25	3.47	4.87	27.56	0.05	76.87
1685.86	624.72	2.93	4.14	32.86	0.24	263.81
1826	995.27	3.4	4.68	39.76	0.05	358.13
12.5	7.12	0.68	2.99	0.37	6.03	3.95
1.25	<1.60	1.29	3.81	4.24	<0.05	0.27
25474.08	24719.03	10131.99	21154.76	10007.85	10445.79	9990.9
4897.31	5040.35	1978.25	3729.11	1993.53	1867.97	2001.89
1057.22	1007.93	407.27	868.71	403.55	409.77	400.04
188.29	194.71	77.56	154.67	78.64	80.31	79.58
41.24	41.52	16.71	31.39	16.37	17.4	16.17
8.5	7.8	3.06	6.59	3.13	2.9	3.17
1.25	1.62	0.67	1.27	0.65	0.68	0.64

IL7	IL8/CXCL8	IL9	IL10	IL12P40	IL12P70	IL13
IL7	IL8/CXCL8	IL9	IL10	IL12P40	IL12P70	IL13
pg/mL	pg/mL	pg/mL	pg/mL	pg/mL	pg/mL	pg/mL
<0.64	<0.64	<0.64	<2.52	0.53	<0.45	<4.14
19.42	>10000.00	<0.64	520.6	0.53	7.32	55.67
20.46	>10000.00	<0.64	524.32	12.09	3.75	61.88
15.88	>10000.00	<0.64	1481.16	15.54	20.82	66.18
19.97	>10000.00	<0.64	1938.46	8.53	19.7	72.86
5.14	1.09	<0.64	<2.52	180.48	12.79	18.43
14.04	<0.64	<0.64	<2.52	0.53	0.45	4.14
10214.55	8363.01	>10000.00	40541.44	98482.99	52103.54	174028.2
1941.53	2305.25	933.18	7801.3	20677.6	9602.46	17340.55
419.03	377.9	425.16	1647.46	4064.53	2118.64	4440.83
76.19	80.43	72.93	316.56	753.01	381.04	769.59
16.7	16.81	20.49	63.25	151.29	82.56	154.67
3.13	3.05	7.66	13.06	55.82	15.87	42.42
0.65	0.66	<0.64	2.52	0.53	3.18	<4.14

IL15	IL17A/CTLA8	IL17E/IL-25	IL17F	IL18	IL22	IL27
IL15 pg/mL	IL17A/CTLA8 pg/mL	IL17E/IL-25 pg/mL	IL17F pg/mL	IL18 pg/mL	IL22 pg/mL	IL27 pg/mL
0.55	<1.04	24.73	1.51	2.39	4.64	41.46
<0.55	4.17	1133.12	5.68	<0.64	165.51	358.96
<0.55	3.81	1241.49	8	<0.64	159.64	358.96
<0.55	3.45	1540.61	6.83	11.58	163.18	358.96
<0.55	4.17	1413.13	9.56	13.76	180.39	332.16
5.27	5.7	2202.45	19.35	426.69	103.74	1710.1
<0.55	1.9	527.55	7.61	<0.64	116.43	194.09
52174.62	21701.84	622032.56	476835.71	10008.98	>200000.00	>250000.00
9587.9	3849.39	128907.42	110686.97	1992.47	16056.34	46265
2074.63	873.6	25234.94	18946.99	406.92	7709.51	10049.71
393.49	147.46	4479.09	3890.44	76.49	1616.24	1996.61
79.54	32.93	1284.59	858.39	18.87	317.09	400.95
16.2	6.91	238.82	154.4	1.1	66.11	70.28
3.18	1.04	<24.73	32.25	1.97	10.82	41.46

IP10/CXCL10	MCP1/CCL2	MCP3/CCL7	MCSF	MDC/CCL22	MIG/CXCL9	MIP1A/CCL3
IP10/CXCL10	MCP1/CCL2	MCP3/CCL7	MCSF	MDC/CCL22	MIG/CXCL9	MIP1A/CCL3
pg/mL	pg/mL	pg/mL	pg/mL	pg/mL	pg/mL	pg/mL
<2.48	<3.20	8.76	<0.10	<0.16	<6.40	4.89
9.42	>50000.00	4538.21	<0.10	26.79	1945.48	>50000.00
7.92	>50000.00	4430.17	<0.10	25.51	1771.19	>50000.00
10.59	>50000.00	671.83	0.1	8.99	2009.87	>50000.00
16.35	>50000.00	3441.93	3.56	13.32	2187.6	>50000.00
21.77	289.19	65.77	418.85	396.48	1464.52	37.7
6.83	<3.20	29.74	<0.10	0.16	<6.40	71.58
87238.26	28799.16	66282.67	667705.57	11369.35	100609.25	>50000.00
6837.55	12824.54	29077.23	123269.23	1954.08	19708.1	6869.7
1553.75	2028.76	5992.81	25269.68	404.34	4371.2	2007.01
329.08	387.06	926.65	4844.38	78.97	720.82	411.77
63.26	82.72	206.94	1060.09	16.22	171.59	74.72
12.91	15.6	39.59	192.17	3.15	40.93	20.66
2.48	3.35	8.07	40.66	0.66	<6.40	<3.20

MIP1B/CCL4	PDGFAA	PDGFAB/BB	RANTES/CCL5	TGFA	TNFA	TNFB/LYMPH
MIP1B/CCL4<br>pg/mL	PDGFAA<br>pg/mL	PDGFAB/BB<br>pg/mL	RANTES/CCL5<br>pg/mL	TGFA<br>pg/mL	TNFA<br>pg/mL	TNFB/LYMPHOTOXINA(LTA)<br>pg/mL
<0.38	<12.80	91.63	0.7	<1.28	<6.40	0.46
>6000.00	329	804.98	1087.73	145.09	1871.8	16.38
>6000.00	313.82	963.33	>20000.00	139.38	1254.71	13.72
>6000.00	270.73	1039.98	>20000.00	275.11	>121373.06	16.67
>6000.00	285.12	1067.62	>20000.00	300.62	>121373.06	20.41
43.16	979.86	10009.74	5808.14	2.24	40.45	3.19
2.06	<12.80	838.49	<0.25	<1.28	<6.40	1.85
>6000.00	>200000.00	162347.53	>20000.00	50179.52	121373.06	25593.87
508.91	32006.37	29497.27	3139.74	2579.92	19361.59	4871.61
244.8	8298.04	6163.86	810.4	878.79	4167.67	1101.86
46.39	1546.87	1143.09	158.04	168.77	757.31	184.25
10.8	329.9	377.43	31.27	31.17	167.71	39.27
3.59	63.01	182.53	8.77	6.44	31.46	8.82
<0.38	12.91	<9.60	0.25	1.28	6.44	1.5

VEGF

VEGF pg/mL
1.45
1840.2
1495.72
1629.34
2021.29
16.02
4.26
40239.41
7895.71
1686
300.81
65.4
13.24
2.44

		SCD40L	EGF	EOTAXIN/CCL	FGF2/FGFB	FLT3L
Group Name	Wells	SCD40L pg/mL	EGF pg/mL	EOTAXIN/CCL11 pg/mL	FGF2/FGFB pg/mL	FLT3L pg/mL
SB	H1,	<12.80	<2.93	2.92	20.18	0.13
Mock (Ultra)	D9,	70.21	13.73	8.66	<20.18	<0.13
Mock (Ultra)	E9,	64.84	12.49	9.62	<20.18	<0.13
Mock (Ultra)	F9,	75.5	14.2	11.13	<20.18	0.13
Eh EVs (Ultra)	G9,	70.21	9.98	10.89	<20.18	<0.13
Eh EVs (Ultra)	H9,	64.84	10.28	11.37	<20.18	<0.13
Eh EVs (Ultra)	A10,	53.79	11.83	11.61	<20.18	<0.13
CONS-4 DX3 (	H3,H2,	298.83	13.13	75.54	<20.18	3.6
PBS	H4,	<12.80	8.69	2.3	<20.18	<0.13
S7	A1,	201868.02	258108.73	97209.41	807366.54	10806.21
S6	B1,	39885.18	10147.89	8823.9	61959.58	3618.37
S5	C1,	8214.64	1863.77	2020.35	17730.79	570.16
S4	D1,	1552.92	415.97	401.03	3103.29	123.54
S3	E1,	312.09	78.22	79.74	651.33	23.78
S2	F1,	85.84	16.42	16.03	126.23	4.79
S1	G1,	<12.80	2.93	3.19	26.46	0.96

FRACTALKINE	GCSF	GMCSF	GROA	IFNA2	IFNG	IL1A
FRACTALKINE/CX3CL1	GCSF	GMCSF	GROA	IFNA2	IFNG	IL1A
pg/mL	pg/mL	pg/mL	pg/mL	pg/mL	pg/mL	pg/mL
<13.67	<3.76	5.91	<0.96	<2.99	0.51	<4.80
<13.67	110.11	2928.67	8.06	<2.99	33.15	2029.73
<13.67	123.9	3656.2	13.62	<2.99	33.15	1994.47
<13.67	165.17	5097.91	14.54	2.99	35.97	2105.78
<13.67	1396.35	22474.81	2.25	39.04	25.63	2536.92
<13.67	774.77	17099.32	4.84	37.58	33.44	2472.05
<13.67	905.16	18886.69	5.21	41.9	34.11	2643.46
<13.67	104.03	<1.70	<0.96	<2.99	2.11	<4.80
<13.67	26.1	37	<0.96	<2.99	0.89	<4.80
376695.03	77590.77	42235.49	47142.42	153845.6	20524.65	51755.22
119717.86	14724.9	7239.44	4009.86	24078.6	3986.88	16803.87
19009.82	3075.79	1676.34	774.8	5091.2	810.56	2662.02
4155.98	589.75	368.94	166.11	1020.98	156.24	620.99
778.36	118.34	44.19	30.53	186.22	32.62	124.74
164.16	26.1	22.72	7.13	47.46	6.39	22.62
30.68	3.76	1.7	0.96	5.79	1.26	4.85

IL1B	IL1RA	IL2	IL3	IL4	IL5	IL6
IL1B<br>pg/mL	IL1RA<br>pg/mL	IL2<br>pg/mL	IL3<br>pg/mL	IL4<br>pg/mL	IL5<br>pg/mL	IL6<br>pg/mL
<1.22	<1.60	0.27	0.34	<0.32	<0.64	<0.64
999.85	556.81	1.74	1.84	36.2	<0.64	186.52
950.47	682.76	1.68	1.75	46.84	<0.64	218.19
1036.87	840.91	1.68	2.07	50.56	<0.64	296.08
2189.9	1171.86	1.29	1.66	28.59	<0.64	425.04
1641.91	1175.47	1.85	1.48	41.88	<0.64	305.84
1682.07	1201.53	1.63	1.25	45.25	<0.64	321.77
<1.22	8.9	<0.27	1.29	0.44	2.97	2.14
<1.22	<1.60	0.73	2.07	2.88	<0.64	<0.64
25446.64	24547.45	10047.2	20273.25	10020.48	10374.33	10131.93
4890.5	5125.92	1991.88	3972.58	1998.79	1905.94	2012.39
1051.49	984.33	400.35	781.95	397.5	391.84	388.2
192.56	200.42	80.95	174.78	81.73	89.89	81.8
39	40.32	15.58	29.19	15.54	15.05	15.73
9.23	7.93	3.33	6.91	3.3	3.22	3.12
1.22	1.61	0.61	1.25	0.63	0.65	0.64

IL7	IL8/CXCL8	IL9	IL10	IL12P40	IL12P70	IL13
IL7	IL8/CXCL8	IL9	IL10	IL12P40	IL12P70	IL13
pg/mL	pg/mL	pg/mL	pg/mL	pg/mL	pg/mL	pg/mL
<0.62	<0.63	<0.64	0.69	6.17	<0.48	<4.29
9.22	5325.73	<0.64	1721.1	0.7	0.48	53.65
7.44	4286.35	<0.64	2180.73	<0.70	<0.48	64.49
8.96	5003.02	<0.64	2658.87	0.7	0.48	74.94
6.71	2543.69	<0.64	2925	<0.70	1.27	30.24
6.91	3769.34	<0.64	2241.58	<0.70	2.98	48.05
5.54	2550.73	<0.64	2247.91	<0.70	1.53	57.77
3.03	<0.63	<0.64	<0.69	84.63	<0.48	<4.29
7.8	<0.63	<0.64	<0.69	<0.70	<0.48	<4.29
10156.77	9174.96	>10000.00	40638.73	99466.71	51219.95	133579.83
1955.9	2131.76	1198.49	7764.01	20212.02	9711.24	18244.04
404.74	383.26	407.42	1647.27	4008.99	2095.17	4226.63
81.01	83.99	77.42	317.83	798.82	390.66	802.26
15.57	15.47	17.41	63.22	151.88	77.85	148.67
3.3	3.26	4.86	13.03	37.1	17.12	39.37
0.62	0.63	<0.64	2.52	5.27	2.98	4.29

IL15	IL17A/CTLA8	IL17E/IL-25	IL17F	IL18	IL22	IL27
IL15	IL17A/CTLA8	IL17E/IL-25	IL17F	IL18	IL22	IL27
pg/mL	pg/mL	pg/mL	pg/mL	pg/mL	pg/mL	pg/mL
0.07	0.53	169.19	<32.00	<0.64	19.45	76.07
<0.07	4.79	843.7	<32.00	4.81	158.64	245.12
<0.07	5.44	802.89	<32.00	5.18	172.95	262.38
<0.07	7.54	1005.88	<32.00	9.08	183.98	262.38
<0.07	<0.53	802.89	<32.00	30.18	145.48	226.36
<0.07	<0.53	679.71	<32.00	23.62	179.62	226.36
<0.07	<0.53	884.4	<32.00	25.44	197.13	256.77
<0.07	<0.53	1252.75	<32.00	367.56	<12.80	1204.42
<0.07	<0.53	169.19	<32.00	<0.64	83.3	<16.00
50955.44	20612.11	622798.41	527216.1	10015.59	>200000.00	177413.45
9845.12	3934.42	127139.7	98886.62	1989.07	15142.05	57228.22
1984.55	821.28	24297.1	18861.53	406.88	6737.15	9782.64
411.92	158.09	5114.4	4208.81	77.29	1653.55	2026.73
77.72	30.91	985.69	796.25	17.63	312.21	391.91
16.34	7.13	213.56	158.69	2.22	78.15	154.5
3.16	1.07	30.32	32.14	1.05	<12.80	<16.00

IP10/CXCL10	MCP1/CCL2	MCP3/CCL7	MCSF	MDC/CCL22	MIG/CXCL9	MIP1A/CCL3
IP10/CXCL10 pg/mL	MCP1/CCL2 pg/mL	MCP3/CCL7 pg/mL	MCSF pg/mL	MDC/CCL22 pg/mL	MIG/CXCL9 pg/mL	MIP1A/CCL3 pg/mL
<2.15	<2.83	5.38	9.44	<0.60	7.16	<3.20
11.72	>50000.00	9240.6	<9.44	16.07	1703.14	3786.31
11.35	>50000.00	11670.91	<9.44	27.98	1783.69	3161.61
11.54	>50000.00	13316.71	<9.44	25.16	2013.91	5895.02
6.98	>50000.00	797.64	25.39	2.51	1072.72	14605.24
7.82	>50000.00	1611.45	17	2.2	2037.04	24345.02
6.04	>50000.00	2328.44	26.92	3.46	2016.99	22870.02
16.17	288.91	54.64	366.27	374.49	1205.87	<3.20
<2.15	<2.83	21.19	<9.44	<0.60	<5.88	56.92
65464.61	29743.06	120744.93	534024.15	9148.35	99720.56	13262.98
8472.42	14133.92	26230.83	136678.28	2060.96	20185.77	40751.93
1456.53	1863.75	4889.6	24003.67	393.28	3921.05	1945.19
335.95	428.39	1008.44	5167.65	81.91	824.55	404.61
62.74	75.75	199.35	985	15.61	155.26	79.01
13.12	17.13	40.06	200.64	3.32	33.57	17.69
2.15	2.83	7.98	40	0.6	5.88	<3.20

MIP1B/CCL4	PDGFAA	PDGFAB/BB	RANTES/CCL5	TGFA	TNFA	TNFB/LYMPH
MIP1B/CCL4	PDGFAA	PDGFAB/BB	RANTES/CCL5	TGFA	TNFA	TNFB/LYMPHOTOXINA(LTA)
pg/mL	pg/mL	pg/mL	pg/mL	pg/mL	pg/mL	pg/mL
3.1	<12.37	<9.60	4.49	<1.22	1.67	0.2
>6000.00	182.27	690.12	1726.59	63	2647.15	22.06
>6000.00	219.23	494.32	1815.47	80.86	3881.99	30.7
>6000.00	231.84	761.73	2082.32	91.66	6060.34	35.71
2722.74	121.2	728.67	1526.65	113.81	63147.37	20.44
>6000.00	185.52	711.73	2573.36	120.27	49198.12	35.51
>6000.00	198.5	753.57	2761.11	138.46	64021.53	41.92
37.47	683.46	7826.67	3451.68	<1.22	35.25	<0.20
<0.38	<12.37	602.98	<0.81	<1.22	<1.67	1.37
>6000.00	338042.97	161762.26	12918.67	101099.28	95040.11	25277.23
1043.09	38690.29	29497.84	4743.23	2665.43	20322.97	4939.17
242.9	8014.38	6122.27	780.51	832.16	3929.48	1021.58
47.72	1626.98	1160.56	162.38	171.68	822.65	201.44
9.76	308.4	304.44	30.88	30.35	156.94	37.62
4.49	66.98	<9.60	7.4	6.71	32.28	8.89
<0.38	12.37	<9.60	0.81	1.22	6.37	1.47

VEGF	EOTAXIN2/C	MCP2/CCL8	BCA1/CXCL1	MCP4/CCL13	I309/CCL1	IL16
VEGF pg/mL	EOTAXIN2/CCL24/MPIF2 pg/mL	MCP2/CCL8 pg/mL	BCA1/CXCL13 pg/mL	MCP4/CCL13 pg/mL	I309/CCL1 pg/mL	IL16 pg/mL
2.01	<1.93	<1.22	0.24	4.2	<0.28	<1.44
2163.39	3063.23	475.99	<0.24	15.3	2281.19	7.93
3281.64	4742.95	847.88	<0.24	20.22	2467.69	21.03
3508.02	4768.85	1076.85	<0.24	20.63	2448.06	21.39
1861.09	886.13	120.25	<0.24	5.42	2298.07	<1.44
3303.95	1902.03	233.93	<0.24	4.85	2467.69	1.44
4105.59	2234.89	306.9	<0.24	8.44	2129.15	<1.44
13.23	106.97	9.28	5.69	19.86	3.48	<1.44
3.44	<1.93	<1.22	<0.24	4.85	<0.28	<1.44
39985.05	10527.6	4196.4	1010.45	11667.23	2002.92	10981.72
8012.97	2399.81	1430.68	245.34	2280.9	496.57	2410.48
1586.67	660.15	308.19	63.58	645.63	132.39	661.37
330.04	149.49	76.24	15.52	155.24	28.78	147.43
60.73	39.44	20.25	3.9	39.07	8.49	40.61
13.95	10.62	4.63	0.99	9.78	2.07	12.02
2.32	1.93	1.49	0.24	2.43	0.28	<1.44

TARC/CCL17	6CKINE/CCL2	EOTAXIN3/CC	LIF	TPO	SCF	TSLP
TARC/CCL17	6CKINE/CCL 21/EXODUS 2	EOTAXIN3/C CL26	LIF	TPO	SCF	TSLP
pg/mL	pg/mL	pg/mL	pg/mL	pg/mL	pg/mL	pg/mL
<0.24	<4.88	<12.21	0.89	7.82	<1.34	0.14
<0.24	56.98	796.37	15.15	<7.82	<1.34	<0.14
<0.24	17.3	883.3	30.02	<7.82	<1.34	<0.14
<0.24	129.2	856.55	36.86	<7.82	<1.34	<0.14
<0.24	108.2	<12.21	240.72	<7.82	<1.34	<0.14
<0.24	96.85	<12.21	216.47	<7.82	<1.34	<0.14
<0.24	<4.88	<12.21	202.55	<7.82	<1.34	<0.14
10.67	668.49	153.3	5.8	20.62	6.26	0.31
<0.24	<4.88	<12.21	2.12	<7.82	<1.34	<0.14
1025.92	19985.22	19885.04	20163.05	50714.56	10003.41	13042.55
241.64	5022.22	13505.38	4916.28	12242.95	2496.79	2172.87
64.9	1236.59	2945.2	1266.83	3229.54	639.64	594.53
15.22	323.64	747.6	314.28	761.32	148.54	164.84
3.96	56.98	259.69	76.69	192.84	41.48	39.8
0.97	71.58	39.4	19.84	54.76	10.71	9.52
0.24	<4.88	<12.21	4.85	9.73	1.34	2.46

IL33/NFHEV(I	IL20	IL21	IL23	TRAIL/TNFSF1	CTACK/CCL27	SDF1A+B/CXC
IL33/NFHEV(MATURE)	IL20	IL21	IL23	TRAIL/TNFSF 10	CTACK/CCL2 7	SDF1A+B/CX CL12
pg/mL	pg/mL	pg/mL	pg/mL	pg/mL	pg/mL	pg/mL
<4.52	52.15	0.41	23.23	0.28	0.06	<12.71
<4.52	<12.21	<0.41	<10.61	<0.28	<0.06	12.71
<4.52	<12.21	0.77	<10.61	<0.28	<0.06	12.71
<4.52	<12.21	<0.41	<10.61	<0.28	<0.06	<12.71
<4.52	<12.21	1.41	<10.61	<0.28	<0.06	<12.71
<4.52	<12.21	0.77	<10.61	<0.28	<0.06	12.71
<4.52	<12.21	1.1	<10.61	<0.28	<0.06	<12.71
<4.52	<12.21	3.29	<10.61	78.58	270	326.29
<4.52	<12.21	<0.41	<10.61	<0.28	<0.06	<12.71
31995.81	52174.87	20813.33	50027.14	10084.59	4987.21	112161.38
4603.57	12164.35	4814.46	12462.9	2451.42	1259.86	23069.7
1338.76	3219.65	1250.49	3157.41	647.25	310.08	6464.88
296.89	755.86	316.39	769.17	150.63	77.59	1547.76
78.69	216.26	78.01	196.95	40.27	20	391.64
20.55	<12.21	19.44	51.14	9.61	4.78	97.57
4.52	37.06	4.9	10.61	2.46	1.23	24.45

ENA78/CXCL5	MIP1D/MIP5,	IL28A/IFNL2	MIF	SICAM1	FASL	LEPTIN
ENA78/CXCL5 pg/mL	MIP1D/MIP5/CCL15 pg/mL	IL28A/IFNL2 pg/mL	MIF pg/mL	SICAM1 pg/mL	FASL pg/mL	LEPTIN pg/mL
<4.26	<11.95	<2.44	<11.78	<61.04	<8.96	2.81
122.65	<11.95	<2.44	1432.76	94129.97	169.63	24.2
264.71	<11.95	<2.44	1793.33	105492.41	177.94	22.16
346.81	<11.95	<2.44	1847.63	116526.35	117.64	26.22
25.21	<11.95	<2.44	961.03	79230.67	93.84	25.21
39.45	<11.95	<2.44	1133.68	88298.2	139.55	27.23
35.64	<11.95	<2.44	1098.95	95894.5	134.51	18.04
184.68	717.19	<2.44	1244.31	641078.56	381.5	10724.97
<4.26	<11.95	<2.44	<11.78	246.47	8.96	30.24
26424.58	53471.17	9975.28	49972.21	249712.95	49833.38	>150000.00
4804.2	12372.98	2588.18	12528.21	62689.66	12641.07	31206.41
1274.22	3072.26	589.58	3106.69	15541.12	3083.19	9705.55
307.14	797.66	163.73	791.61	3892.89	797.49	2633.53
79.16	191.61	45.09	191.72	1007.56	187.89	486.93
19.58	49.85	12.71	50.17	216.68	51.44	198.74
4.26	11.95	<2.44	11.78	80.36	11.8	34.23

HGF	SFAS/TNFRSF	RESISTIN	SVCAM1	PAI1/SERPINE
HGF pg/mL	SFAS/TNFRSF6 pg/mL	RESISTIN pg/mL	SVCAM1 pg/mL	PAI1/SERPINE pg/mL
<6.46	5.1	139.59	<7.89	<36.58
6.46	28.29	<24.41	7.89	11642.76
23.68	59.9	<24.41	179.56	13488.15
15.7	49.59	<24.41	128.76	13784.84
<6.46	89.97	<24.41	363.32	10909.55
11.32	59.9	<24.41	274.31	13784.84
6.46	44.36	<24.41	274.31	13885.26
1244.97	9551.72	<24.41	1788714.22	41870.29
<6.46	<5.10	<24.41	128.76	<36.58
99935.61	50848.92	99613.93	248155.31	134226.97
25032.15	12729.5	25038.16	64918.36	42199.57
6227.56	2983.86	6148.85	14818.1	8937.51
1577.25	767.11	1711.21	3959.16	2392.23
385.03	203.22	307.17	1059.66	581.75
99.83	59.9	73.46	179.56	146.85
23.68	5.1	53.4	101.99	36.58
